# Construction of an interpretable model for predicting survival outcomes in patients with middle to advanced hepatocellular carcinoma (≥5 cm) using lasso-cox regression

**DOI:** 10.3389/fphar.2024.1452201

**Published:** 2024-09-19

**Authors:** Han Li, Bo Yang, Chenjie Wang, Bo Li, Lei Han, Yi Jiang, Yanqiong Song, Lianbin Wen, Mingyue Rao, Jianwen Zhang, Xueting Li, Kun He, Yunwei Han

**Affiliations:** ^1^ Department of Oncology, The Affiliated Hospital of Southwest Medical University, Luzhou, China; ^2^ Department of General Surgery (Hepatobiliary Surgery), The Affiliated Hospital of Southwest Medical University, Luzhou, China; ^3^ Department of Oncology, Affiliated Hospital of Jining Medical University, Jining, China; ^4^ Sichuan Cancer Hospital & Institute, Sichuan Cancer Center, School of Medicine, University of Electronic Science and Technology of China, Chengdu, China; ^5^ Sichuan Provincial People’s Hospital, Chengdu, China; ^6^ Department of Oncology, 363 Hospital, Chengdu, China; ^7^ Clinical Medical College, Southwest Medical University, Luzhou, China

**Keywords:** LASSO-COX, nomogram, interpretable, hepatocellular carcinoma, radiotherapy

## Abstract

**Background:**

In this retrospective study, we aimed to identify key risk factors and establish an interpretable model for HCC with a diameter ≥ 5 cm using Lasso regression for effective risk stratification and clinical decision-making.

**Methods:**

In this study, 843 patients with advanced hepatocellular carcinoma (HCC) and tumor diameter ≥ 5 cm were included. Using Lasso regression to screen multiple characteristic variables, cox proportional hazard regression and random survival forest models (RSF) were established. By comparing the area under the curve (AUC), the optimal model was selected. The model was visualized, and the order of interpretable importance was determined. Finally, risk stratification was established to identify patients at high risk.

**Result:**

Lasso regression identified 8 factors as characteristic risk factors. Subsequent analysis revealed that the lasso-cox model had AUC values of 0.773, 0.758, and 0.799, while the lasso-RSF model had AUC values of 0.734, 0.695, and 0.741, respectively. Based on these results, the lasso-cox model was chosen as the superior model. Interpretability assessments using SHAP values indicated that the most significant characteristic risk factors, in descending order of importance, were tumor number, BCLC stage, alkaline phosphatase (ALP), ascites, albumin (ALB), and aspartate aminotransferase (AST). Additionally, through risk score stratification and subgroup analysis, it was observed that the median OS of the low-risk group was significantly better than that of the middle- and high-risk groups.

**Conclusion:**

We have developed an interpretable predictive model for middle and late HCC with tumor diameter ≥ 5 cm using lasso-cox regression analysis. This model demonstrates excellent prediction performance and can be utilized for risk stratification.

## Introduction

Hepatocellular carcinoma (HCC), a leading malignancy globally, typically affects individuals with genetic susceptibility who have been exposed to risk factors. Notably, there are significant individual differences in HCC development. Currently, the etiology of HCC is gradually shifting from viral to non-viral origins (Toh et al., 2023).

According to NCCN, the treatment of HCC involves local and systemic therapies. Local regional therapy plays a leading role in the management of HCC in approximately 50%–60% of cases. Radiofrequency ablation (RFA) is the main method for early local treatment, while transarterial chemoembolization (TACE) remains the standard treatment for mid-stage HCC (Llovet et al., 2021). With increasing clinical applications, radiotherapy (RT) has emerged as a reliable and effective treatment option for HCC, depending on the disease severity and patient characteristics. For patients with resectable, single-small hepatitis B virus-associated HCC, there is a high risk of microvascular invasion (MVI), and the response rate to neoadjuvant radiotherapy is promising, with disease-free survival rates of 86.7%, 76.7%, and 60.0% in the first, second, and third years, respectively (Wei et al., 2023). In fact, large hepatocellular carcinoma> 5 cm traditionally requires large hepatectomy. Minor hepatectomy is usually performed with the aim of reducing morbidity and mortality. In NCCN guidelines, non-diffuse HCC with tumor diameter over 5 cm is an indication for external radiotherapy and TACE. Studies from Su suggest that external radiotherapy is better as the primary local treatment for HCC with tumor diameter over 5 cm, especially HCC with tumor diameter 5–10 cm. This discovery laid the foundation for future research on radiotherapy for HCC(4). Drawing from the achievements of the SHARP trial and IMbrave150 trial (Llovet et al., 2008; Liu et al., 2019; Finn et al., 2020), The systemic therapy for HCC has evolved from single-agent targeted therapy (such as sorafenib and lenvatinib) to combination therapy with checkpoint inhibitors (such as atezolizumab combined with bevacizumab) (Yang et al., 2023), and has been recognized by food and Drug Administration (FDA). The updated efficacy and safety data from the IMbrave150 trial demonstrate that the median overall survival (OS) in the atezolizumab combined with bevacizumab group was 19.2 months (95% CI, 17.0–23.7 months), compared to 13.4 months (95% CI, 11.4–16.9 months) in the sorafenib group (Cheng et al., 2022). Previous research has demonstrated that percutaneous acetic acid (PAAI) and RFA are equally effective in the treatment of small (≤5 cm) HCC. However, for HCC tumors ≥ 5 cm, RT and TACE are potential local treatment options. Nevertheless, the optimal local treatment for this tumor size remains controversial. According to Su’s study, the median overall survival (mOS) of the EBRT group was significantly longer than that of the TACE group before and after matching for HCC tumors ≥ 5 cm (14.9 vs 12.3 months, and 16.8 vs 11.4 months, respectively) (Su et al., 2023a).

The prognosis of HCC is complex due to the competing risks of potential cirrhosis and other malignancies. Chronic fibrotic liver disease due to viral or metabolic factors significantly raises HCC risk and often predicts a poorer prognosis (Fujiwara et al., 2018). HCC ranks fifth among cancers in Asia, serving as the second-largest cause of cancer-related deaths, with 72.5% of cases occurring there in 2020 (Zhang et al., 2022). Hepatocellular carcinoma (HCC), prevalent among liver cancers, dominates morbidity and mortality. Key prognostic factors for HCC encompass extrahepatic metastasis and liver function reserves, notably albumin and bilirubin levels (Ho et al., 2023; Schütte et al., 2020). Postoperative HAIC + FOLFOX enhanced DFS in MVI-HCC patients with tolerable side effects. External radiotherapy outcomes for MVI-HCC were unfavorable, but SBRT showed promise, especially in combination with sorafenib. Neoadjuvant radiotherapy yielded favorable responses with low toxicity in resectable, small HBV-HCCs (<5 cm) predicted to have high MVI risk (Wei et al., 2023; Munoz-Schuffenegger et al., 2021). In addition to tumor burden, liver function, and overall physical status, other factors such as tumor biopsy results, serum markers, and more, play significant roles in determining the prognosis of patients with HCC (Kulik and El-Serag, 2019). To establish an effective diagnosis and treatment model for HCC, it is essential to develop risk stratification algorithms and biomarkers to identify individuals at high risk. Additionally, advancements in imaging and blood-based monitoring tests are needed to improve the sensitivity and specificity of early HCC detection. Beyond clinical validation, data are necessary to assess clinical utility, aiming to enhance early tumor detection and ultimately reduce HCC-related mortality (Singal et al., 2023). The assessment of treatment response using the modified Solid Tumour Response Assessment Criteria (mRECIST) category indicated that mRECIST criteria and tumor size could effectively distinguish between patients with a high or low likelihood of recurrence following HCC transplantation (Kim et al., 2014). Cohort studies utilizing serum HCC markers have demonstrated that patients with HCC tumors ≥ 5 cm treated with different interventions exhibit variable survival outcomes. When all three HCC markers are positive, specific interventions can independently predict poor prognosis (Ueno et al., 2015).

In numerous HCC models, tumor size is recognized as a significant predictor of HCC patient prognosis. In some investigations, larger tumors have been associated with increased aggression and progression (Carr and Guerra, 2016; Yan et al., 2020). This means that patients with HCC tumors ≥ 5 cm often have advanced staging. Large HCC face poor prognosis due to expansion and tissue compression, often linked to delayed liver recovery and higher recurrence. Despite this, research on optimal predictive indices and interpretable ML models remains scarce. This study aims to innovatively address this gap, enhancing our understanding of survival risks in large HCCs through advanced machine learning methods. Currently, there is no established clinical predictive model for HCC tumors of this size, hence the development of an interpretable model for advanced patients with HCC tumors ≥ 5 cm undergoing comprehensive treatment (Pawlik et al., 2005).

### Method and patients

The study included 843 patients from three tertiary hospitals in China from May 2017 to December 2023. Inclusion criteria include: a) patients with hepatocellular carcinoma diagnosed pathologically or clinically; b) patients who did not receive any antineoplastic therapy before admission; c) blood routine, biochemistry, electrolytes, and coagulation function measured within 7 days before registration; d) complete clinical data; e) tumordiameter ≥ 5 cm. We excluded patients with other types of malignant tumors. This study was approved by the affiliated Hospital of Ethics Committee of Southwest Medical University (KY2020254). The Ethics Committee abandoned the informed consent form because it was a retrospective study. The data were analyzed anonymously.

### Data acquisition

The collected patient baselines encompassed the following: 1) Serum laboratory indicators: white blood cell (WBC) count, alpha-fetoprotein (AFP), platelet count (PLT), and prothrombin time (PT). 2) Liver function markers: alanine aminotransferase (ALT), aspartate aminotransferase (AST), alkaline phosphatase (ALP), total bilirubin (TBIL), albumin (ALB), and Child-Pugh score. 3) Tumor burden: portal vein tumor thrombus (PVTT), ascites, extrahepatic metastasis, tumor number, and lymph node metastasis. 4) Tumor staging: Barcelona Clinic Liver Cancer (BCLC) staging system. 5) Additional data: gender, age, hepatitis B virus (HBV) status, diabetes mellitus, alcohol consumption, hypertension, and treatment options. OS was defined as the duration from the start of treatment until death or the last follow-up.

### Statistical analysis

All data were analyzed by SPSS26.0 (SPSS, Chicago, Illinois, United States) and R4.2.2 software. Bilateral *P* < 0.05 is considered to be statistically significant. For classified variables (χ-2 test). Two predictive models (cox and Random Survival Forest (RSF)) were established, including Lasso regression to screen prognostic characteristics. Finally, the risk score is established based on nomogram, and the cutoff value is determined by X-tile tool; the mOS is estimated by Kaplan-Meier statistics, and the results are compared by Log-rank test. Lasso outperforms Ridge in feature selection occasionally, zeroing out unimportant coefficients via L1 regularization. Ideal for large, irregular feature sets, Lasso generates sparse models with few non-zero coefficients, simplifying and enhancing interpretability. This conciseness is crucial for clinical grafting in medical models, making Lasso the preferred choice.

## Result

### Patient characteristics

Among the 843 patients included, there were 709 males, 411patients with HBV infection, 298patients with ascites diagnosed by imaging, 572patients with multiple tumors, 396patients with portal vein thrombosis and 240patients with BCLC stage B. 354 cases had lymph node metastasis, 183cases had distant metastasis. The specific baseline situation is shown in Table 1.

**TABLE 1:** Baseline characteristics of patients.

Variable	Total (n = 843)	Group		P
		1 (n = 590)	0 (n = 253)	
BCLC, n (%)				0.014
B	240 (28.47)	151 (25.59)	89 (35.18)	
C	556 (65.95)	407 (68.98)	149 (58.89)	
D	47 (5.58)	32 (5.42)	15 (5.93)	
PVTT, n (%)				0.668
No	447 (53.02)	310 (52.54)	137 (54.15)	
Yes	396 (46.98)	280 (47.46)	116 (45.85)	
Number, n (%)				0.068
<3	271 (32.15)	201 (34.07)	70 (27.67)	
≥3	572 (67.85)	389 (65.93)	183 (72.33)	
N, n (%)				0.27
No	489 (58.01)	335 (56.78)	154 (60.87)	
Yes	354 (41.99)	255 (43.22)	99 (39.13)	
M, n (%)				0.475
No	660 (78.29)	458 (77.63)	202 (79.84)	
Yes	183 (21.71)	132 (22.37)	51 (20.16)	
Child-pugh, n (%)				0.92
A	508 (60.26)	353 (59.83)	155 (61.26)	
B	285 (33.81)	202 (34.24)	83 (32.81)	
C	50 (5.93)	35 (5.93)	15 (5.93)	
Ascites, n (%)				0.589
No	545 (64.65)	378 (64.07)	167 (66.01)	
Yes	298 (35.35)	212 (35.93)	86 (33.99)	
Age, n (%)				0.523
<60	516 (61.21)	357 (60.51)	159 (62.85)	
≥60	327 (38.79)	233 (39.49)	94 (37.15)	
Gender, n (%)				0.965
Female	134 (15.9)	94 (15.93)	40 (15.81)	
Male	709 (84.1)	496 (84.07)	213 (84.19)	
Hyper, n (%)				0.252
No	683 (81.02)	484 (82.03)	199 (78.66)	
Yes	160 (18.98)	106 (17.97)	54 (21.34)	
Hbv, n (%)				0.583
No	432 (51.25)	306 (51.86)	126 (49.80)	
Yes	411 (48.75)	284 (48.14)	127 (50.20)	
Smoke, n (%)				0.862
No	437 (51.84)	307 (52.03)	130 (51.38)	
Yes	406 (48.16)	283 (47.97)	123 (48.62)	
Ancohol, n (%)				0.695
No	505 (59.91)	356 (60.34)	149 (58.89)	
Yes	338 (40.09)	234 (39.66)	104 (41.11)	
Wbc, n (%)				0.484
<4*10^9/L	100 (11.86)	73 (12.37)	27 (10.67)	
≥4*10^9/L	743 (88.14)	517 (87.63)	226 (89.33)	
Hb, n (%)				0.748
<100 g/L	138 (16.37)	95 (16.10)	43 (17.00)	
≥100 g/L	705 (83.63)	495 (83.90)	210 (83.00)	
Plt, n (%)				0.89
<100*10^9/L	194 (23.01)	135 (22.88)	59 (23.32)	
≥100*10^9/L	649 (76.99)	455 (77.12)	194 (76.68)	
Alt, n (%)				0.213
<40U/L	389 (46.14)	264 (44.75)	125 (49.41)	
≥40U/L	454 (53.86)	326 (55.25)	128 (50.59)	
Ast, n (%)				0.991
<40U/L	263 (31.2)	184 (31.19)	79 (31.23)	
≥40U/L	580 (68.8)	406 (68.81)	174 (68.77)	
Alb, n (%)				0.238
<35 g/L	308 (36.54)	208 (35.25)	100 (39.53)	
≥35 g/L	535 (63.46)	382 (64.75)	153 (60.47)	
Tbil, n (%)				0.404
<25 umol/L	448 (53.14)	308 (52.20)	140 (55.34)	
≥25 umol/L	395 (46.86)	282 (47.80)	113 (44.66)	
Alp, n (%)				0.167
<125U/L	306 (36.3)	223 (37.80)	83 (32.81)	
≥125U/L	537 (63.7)	367 (62.20)	170 (67.19)	
Pt, n (%)				0.493
<12S	129 (15.3)	87 (14.75)	42 (16.60)	
≥12S	714 (84.7)	503 (85.25)	211 (83.40)	
Afp, n (%)				0.479
<200 ng/L	447 (53.02)	306 (51.86)	141 (55.73)	
200–400 ng/L	53 (6.29)	40 (6.78)	13 (5.14)	
≥400 ng/L	343 (40.69)	244 (41.36)	99 (39.13)	

**Abbreviations**: BCLC, Barcelona Clinic Liver Cancer;PVTT, portal vein tumor thrombus; HBV, hepatitis B virus; Alb, Albumin; ALP, alkaline phosphatase; AFP, alpha-fetoprotein; ALT, alanine aminotransferase; Pt, Prothrombin time.

### Prediction model based on lasso-cox regression

**FIGURE 1:**
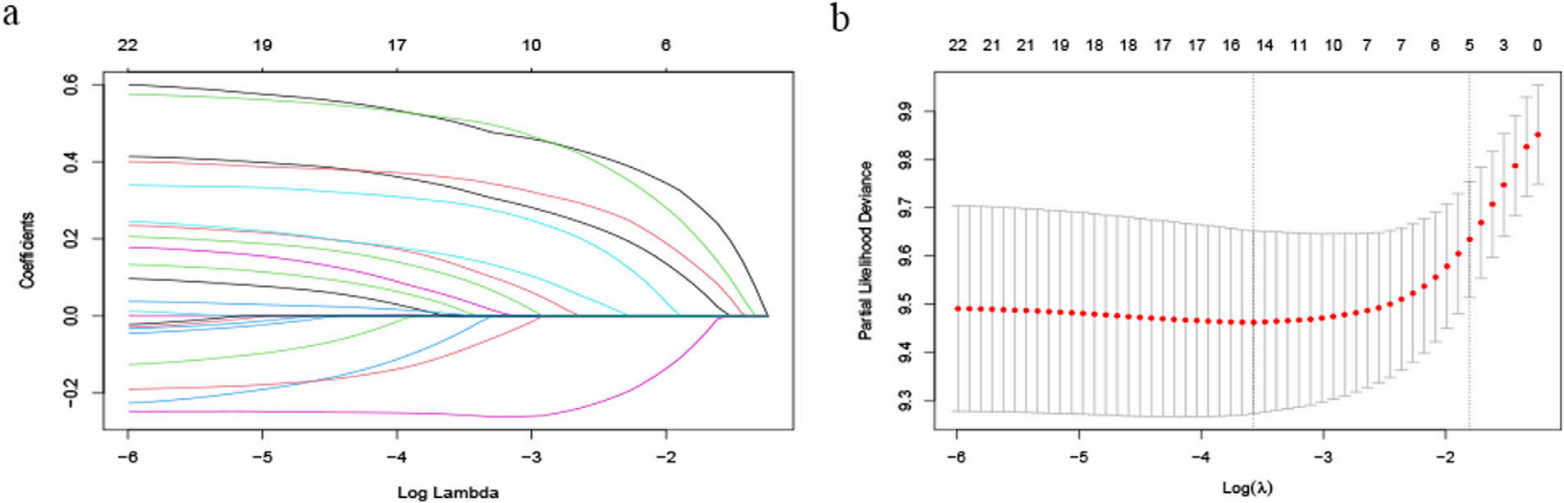
Variable Screening Based on Lasso Regression: **(A)** Characteristics of Variable Coefficients and **(B)** Cross-validation Procedure for Optimal λ Selection.

**FIGURE 2:**
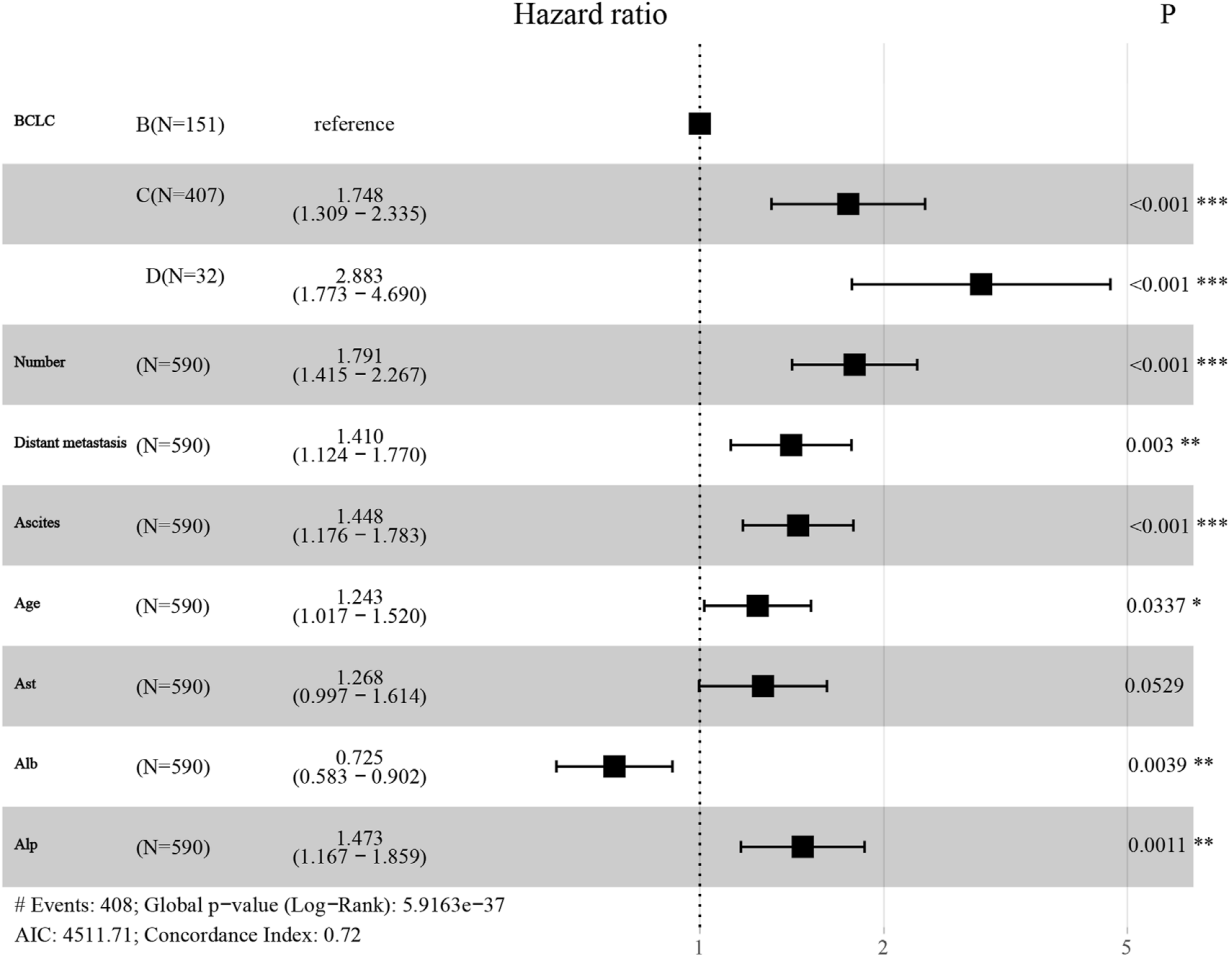
Cox proportional hazard regression, used to predict OS based on Lasso regression.

**FIGURE 3:**
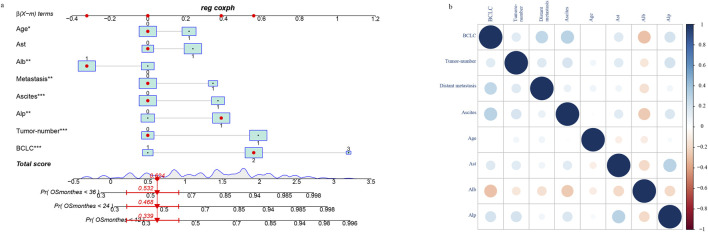
Construction of nomogram based on lasso-cox regression **(A)** and correlation of characteristic variables **(B)**.

We enrolled patients from May 2017 to December 2023 and divided them into a training set and a verification set at a ratio of 7:3. The training set was utilized to screen features and establish models, utilizing lasso regression to determine the parameters. The variation characteristics of the coefficients of these variables are presented in Figure 1A. Iterative analysis was conducted using cross-validation (Figure 1B). When λ = min, the variables were included as feature variables. Among them, BCLC staging, tumor number, distant metastasis, ascites, age ≥60, aspartate aminotransferase ≥40 ug/L, albumin <35 g/L, alkaline phosphatase ≥125 ug/L were all identified as characteristic factors. Based on the features selected by lasso regression, a multi-factor Cox regression model (Figure 2) was further established. A nomogram visualizes our cox model (Figure 3A), and the correlation heat map shows the correlation between features (Figure 3B).

**FIGURE 4:**
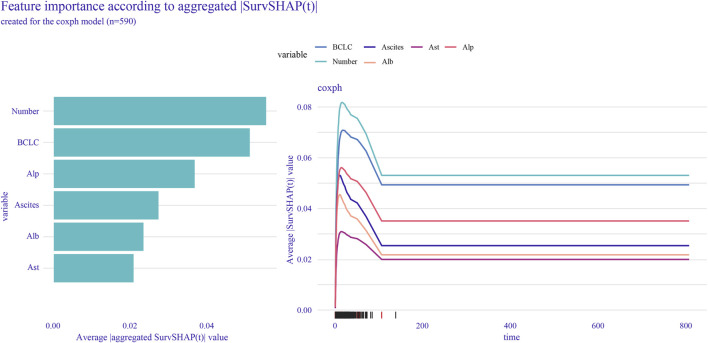
SurvSHAP was used to calculate feature importance and rank the top six features selected by lasso-cox.

To further assess the specific impact of each feature variable as a risk factor on OS, we employed the global interpretation method to construct SurvSHAP. This approach quantifies the contribution of each feature to the model and identifies the prediction contribution of the model to a broader range of outcomes. Using SurvSHAP, we calculated the average SHAP value of each feature across all samples and presented the top six risk factors in lasso-cox (Figure 4).

### Prediction model based on RSF

**FIGURE 5:**
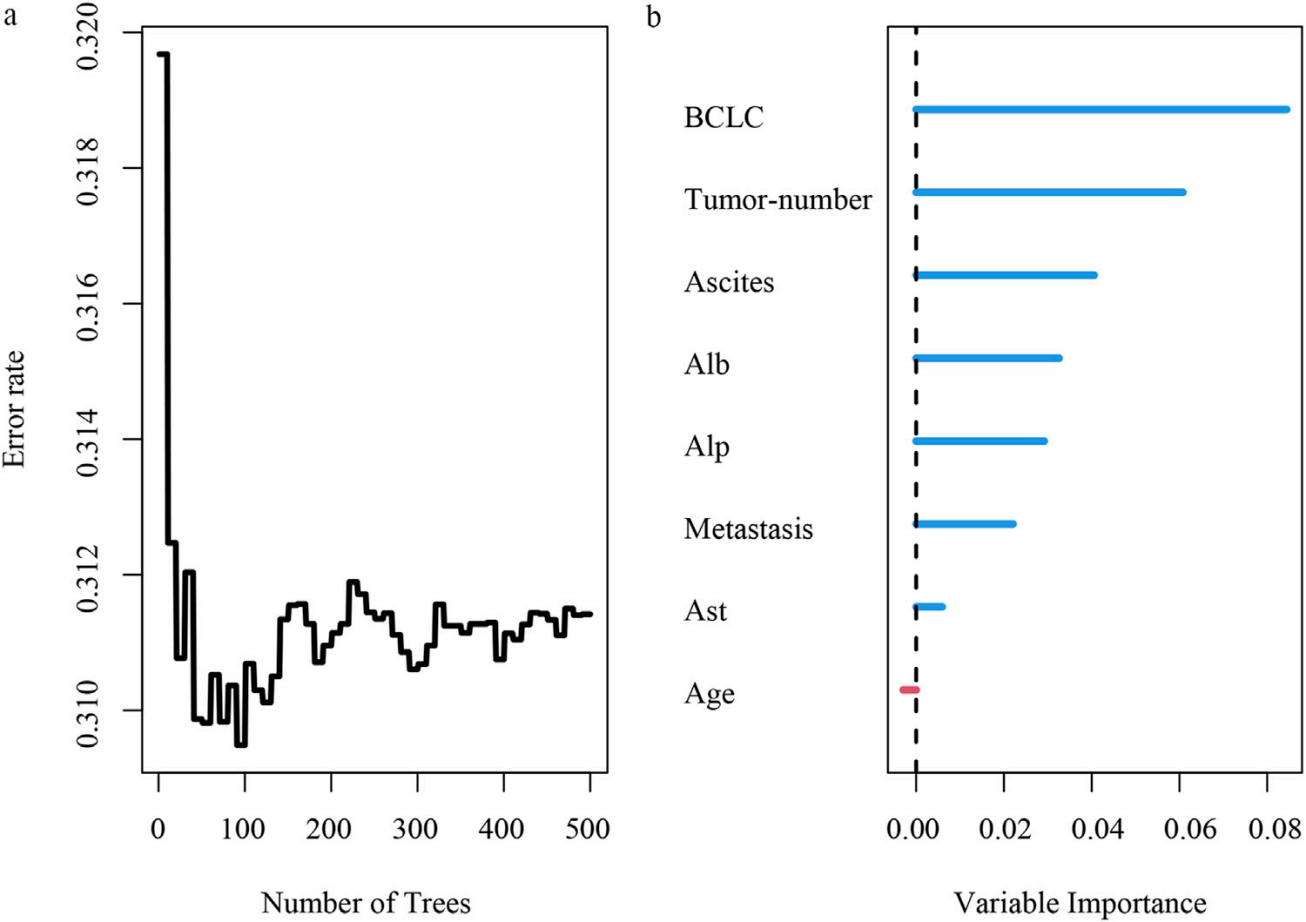
Risk analysis of HCC recurrence based on random survival forest. **(A)** error rate of random survival forest; **(B)** importance ranking of variables.

By using the features selected via lasso regression, we constructed the RSF model and visualized its feature importance ranking (Figures 5A, B). Through meticulous parameter tuning, we observed that when ntree is set to 200, the error rate of the model tends to stabilize.

### Model verification

**FIGURE 6:**
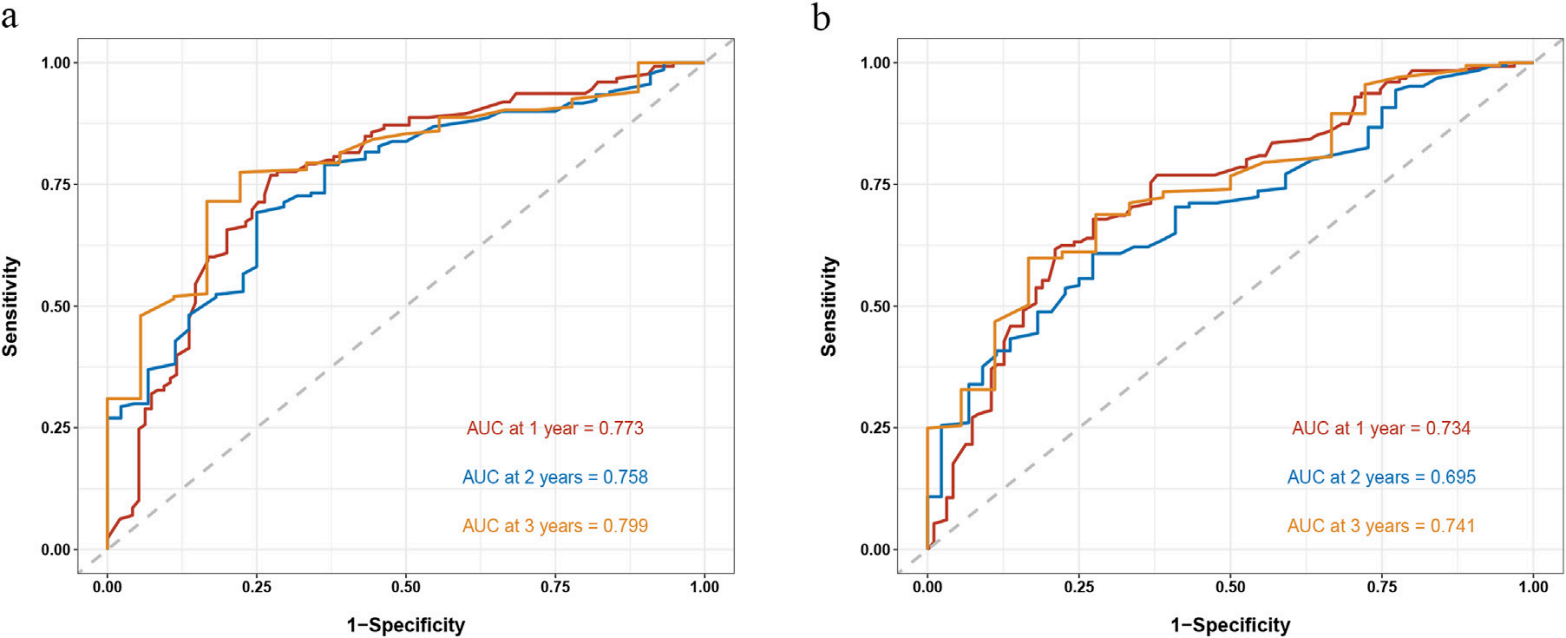
TimeROC of cox model **(A)** and RSF model **(B)** after lasso variable filtering.

The model is verified in the verification set. We establish the timeROC curve under the condition of lasso-cox and lasso-RSF respectively. For the lasso-cox model, the AUC values are 0.773, 0.758, and 0.799 for 1-year, 2-year, and 3-year predictions, respectively,for the lass-RSF model, the AUC values are 0.734, 0.695, and 0.741 for 1-year, 2-year, and 3-year predictions, respectively (Figures 6A, B).

### Risk stratification

**FIGURE 7:**
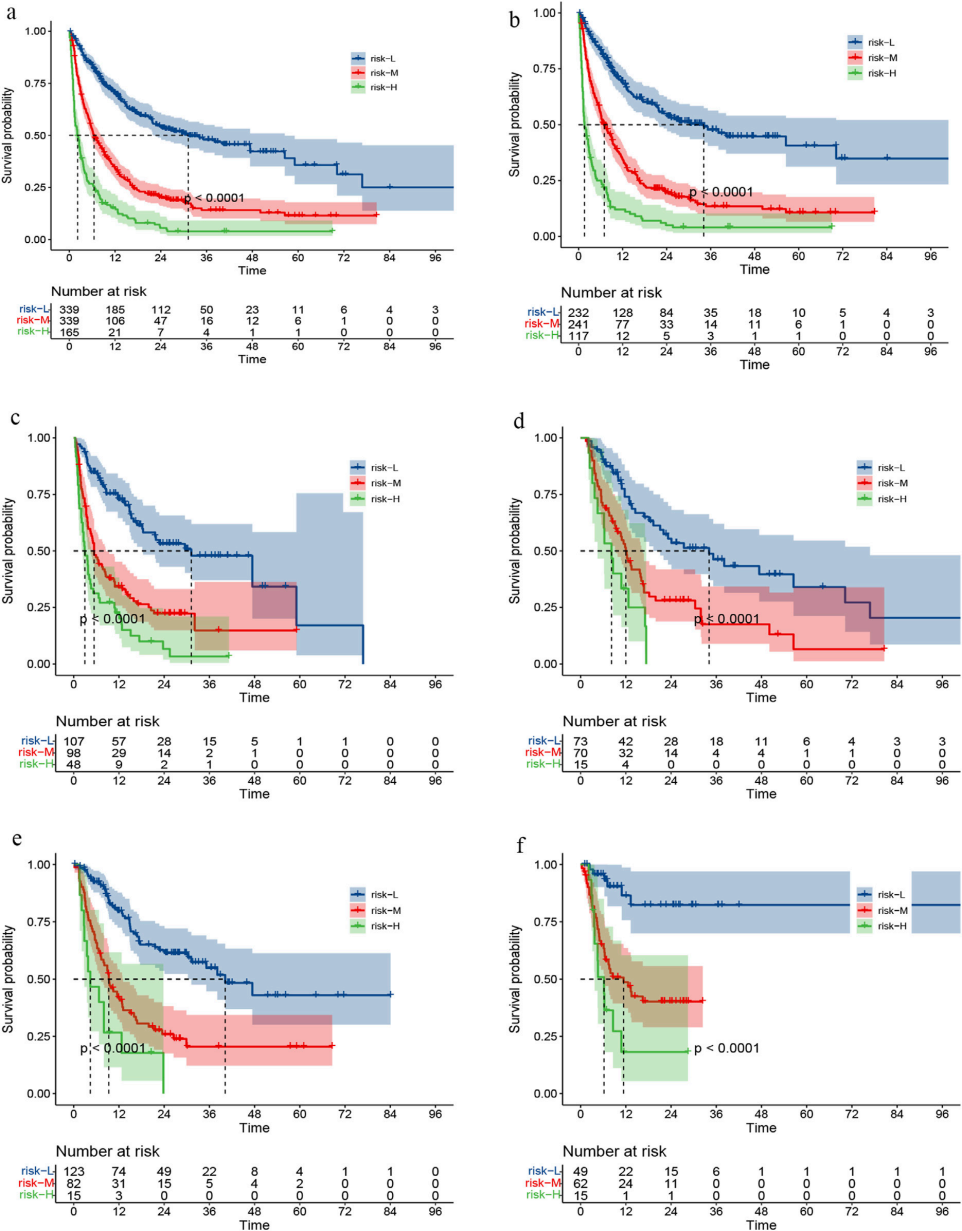
Survival curve under risk stratification corresponding to overall cohort **(A)**, training set **(B)**, validation set **(C)**, RT set **(D)**, TACE set **(E)** or targeted combined immunotherapy set **(F)**, respectively.

We calculated the risk score for all patients based on the nomogram and utilized the X-tile software to identify the optimal risk stratification demarcation point for dividing the sample into low-risk, middle-risk, and high-risk groups (Supplementary Figure S1). Notably, distinct risk scores were associated with significantly different survival outcomes (Figure 7A).

In the subgroup analysis, we compared survival outcomes across different risk categories between the training and validation datasets (Figures 7B, C). We also evaluated patients who RT,TACE, or targeted combined immunotherapy (TI) as part of their treatment, not only those who received these specific therapies. This enabled us to assess survival disparities among patients at high, medium, and low risk within these three groups (Figures 7D–F).

## Discussion

The survival prognosis for patients with HCC is bleak; however, tumor progression can be monitored by tracking characteristic risk factors. Improving the identification of tumor-specific characteristics can facilitate the implementation of radical treatment (Hong et al., 2018). Since the last official update of BCLC’s HCC prognosis and treatment strategy in 2018, significant progress has been made in the field of HCC treatment. However, the data available for selecting interventions remains insufficiently mature to be included in evidence-based models used by clinicians and researchers. To address this issue, it is imperative to establish models that can determine different clinical prognoses based on various characteristics (Reig et al., 2022).

The study enrolled 843 patients and developed a nomogram, utilizing lasso regression to screen several variables. This established model comprises eight indices for clinical application: BCLC staging, tumor number, presence of distant metastasis, ascites, age ≥60 years, aspartate aminotransferase ≥40 ug/L, albumin <35 g/L, and alkaline phosphatase ≥125 ug/L. Drawing from the characteristic factors identified by Lasso screening, we developed cox and RSF models and compared them in terms of receiver operating characteristic (ROC) and c-index values. We also ranked feature importance using SHAP value or variable importance in projection (VIMP) in these two models. The prediction model based on lasso-cox regression exhibited good performance in predicting 1-, 2-, and 3-year survival. Finally, patients were clearly stratified into three risk groups based on survival, revealing significant survival benefits in the low-risk group compared with the high-risk group.

The BCLC staging system provides clear guidance for prognosis and treatment. Stage B includes asymptomatic patients with multinodular HCC, while Stage C includes patients with symptomatic or invasive tumors. Patients in Stages B and C may potentially benefit from novel treatment approaches investigated in Phase II studies or randomized controlled trials. The prognosis for Stage D patients is exceedingly poor, and the treatment options are more limited (Llovet et al., 1999). The study of tumor diversity has revealed that the genomic characteristics of liver cancer in some patients cannot be fully captured through sequencing individual lesions. Comparative genomic analysis of multiple lesions associated with liver cancer offers valuable insights into the genetic alterations linked to tumor progression. This suggests that a higher number of tumor lesions may carry more prognostic information, emphasizing the importance of studying large tumors (≥5 cm) or multiple tumors (Xue et al., 2016; Lackner et al., 2019). HCC patients with significant ascites exhibited signs of peritoneal infiltration, positive cytological examination results, and elevated fibronectin levels (Martins et al., 2006). Tumor-associated ascites can significantly impact the baseline liver function, and ascites volumes exceeding 500 mL have a notable impact on liver reserve and tumor status (Zhang et al., 2009), Therefore, it can serve as a predictor of survival prior to treatment. Additionally, numerous studies have established tumor number, ascites, age, and distant metastasis as independent risk factors for tumor prognosis, a fact that is widely accepted (Nouso et al., 2010). AST and ALT, primarily in liver cells, elevate in serum upon cell damage. Highly sensitive to liver injury, they’re key indicators for assessing liver health and diagnosing disease. In HCC, their rising levels mirror liver cell deterioration, reflecting disease progression.

Studies have demonstrated that the change in ALP levels is an independent prognostic factor in HCC following partial hepatectomy. ALP elevation serves as a functional biomarker of liver function, rather than a specific biomarker for HCC. The higher regenerative capacity may be associated with the increase in ALP levels postoperatively (Huang et al., 2022). Furthermore, the nomogram utilizing lactate dehydrogenase (LDH) and ALP demonstrates good predictive accuracy for HCC. For HCC patients with elevated ALP or ALP/LDH levels, close monitoring and consideration of adjuvant treatment should be considered (Su et al., 2023b). Wu’s study also revealed that ALP levels can predict the prognosis of patients with HBV-related HCC following radical hepatectomy (Wu et al., 2016). Monitoring these enzymes aids in understanding HCC status and optimizing treatment. ALP, ubiquitous in liver, bone, gut, kidney, and placenta, is an enzyme expelled by the liver to the gallbladder. Elevated serum ALP often signals hepatobiliary disorders, as liver is its primary source in blood.

The Lasso-cox regression seamlessly integrates the strengths of both lasso and cox regressions, ensuring precision in high-dimensional data analysis. By leveraging lasso’s variable selection, the model eliminates unnecessary variables and retains crucial predictors of survival time, yielding a concise yet powerful cox model. This refined approach consistently delivers enhanced accuracy in predicting individual survival durations. Lasso regression mitigates the issue of multi-collinearity among variables, and variable screening significantly reduces overfitting. This study demonstrates that the RSF model exhibits inferior AUC values compared to the Lasso-Cox model. Therefore, a nomogram based on the Lasso-Cox regression model is developed, enhancing the explanation of feature importance using SHAP values. This offers medical professionals valuable insights for intuitive analysis of individual prognostic risks, facilitating the identification of high-risk patients in clinical settings. However, there are limitations to this study. Firstly, the study focuses on HCCs ≥5 cm; future research should explore HCC subtypes between 5 and 10 cm and ≥10 cm. Secondly, more centers are needed to verify the generalizability of external validation datasets. Lastly, future multicenter studies with larger sample sizes are necessary to validate and disseminate these findings in a larger population.

## Conclusion

We have developed an interpretable predictive model for middle and late HCC with tumor diameter ≥ 5 cm using lasso-cox regression analysis. This model demonstrates excellent prediction performance and can be utilized for risk stratification.

## Data Availability

The raw data supporting the conclusions of this article will be made available by the authors, without undue reservation.
