# Supplementary figures and images for: Construction of an interpretable model for predicting survival outcomes in patients with middle to advanced hepatocellular carcinoma (≥5 cm) using lasso-cox regression
Source: Front Pharmacol. 2024 Sep 19;15:1452201. doi: 10.3389/fphar.2024.1452201 (PMC11450703; doi:10.3389/fphar.2024.1452201)

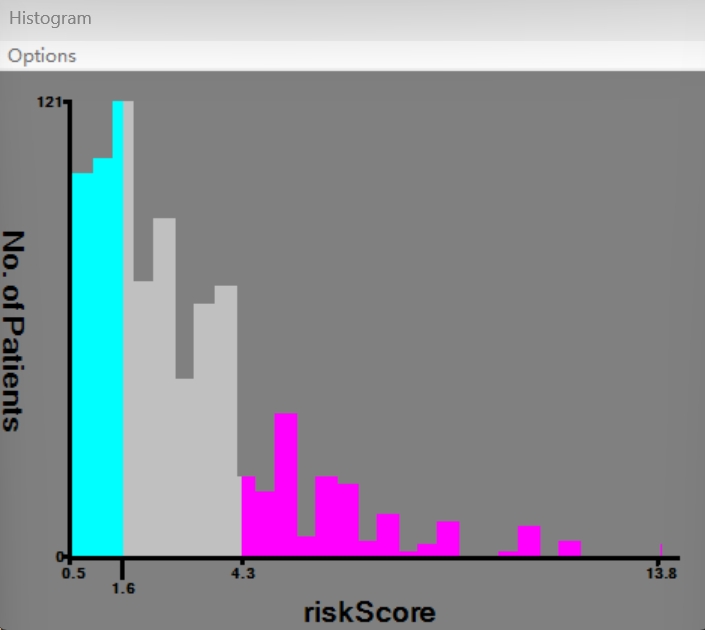


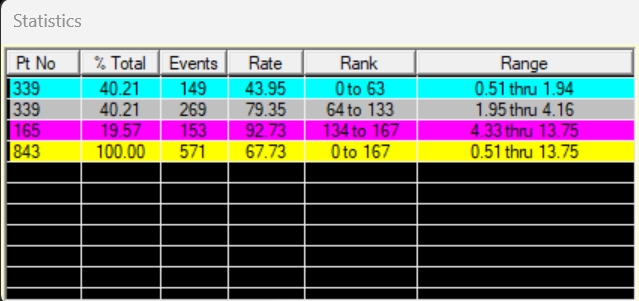

Supplement: Supplementary file 1 [file Table1.DOCX]
